# Real-Time Adaptive Control of a Magnetic Levitation System with a Large Range of Load Disturbance

**DOI:** 10.3390/s18051512

**Published:** 2018-05-11

**Authors:** Zhizhou Zhang, Xiaolong Li

**Affiliations:** 1 College of Aerospace Science, National University of Defense Technology, Changsha 410073, China; 2 College of Intelligence Science, National University of Defense Technology, Changsha 410073, China; lixiaolong@maglev.cn

**Keywords:** magnetic levitation system, load disturbance, fast current loop, real-time adaptive control, stability

## Abstract

In an idle light-load or a full-load condition, the change of the load mass of a suspension system is very significant. If the control parameters of conventional control methods remain unchanged, the suspension performance of the control system deteriorates rapidly or even loses stability when the load mass changes in a large range. In this paper, a real-time adaptive control method for a magnetic levitation system with large range of mass changes is proposed. First, the suspension control system model of the maglev train is built up, and the stability of the closed-loop system is analyzed. Then, a fast inner current-loop is used to simplify the design of the suspension control system, and an adaptive control method is put forward to ensure that the system is still in a stable state when the load mass varies in a wide range. Simulations and experiments show that when the load mass of the maglev system varies greatly, the adaptive control method is effective to suspend the system stably with a given displacement.

## 1. Introduction

A low-speed maglev train uses an electromagnetic attractive force to support the car body, which has the advantages of little noise, a small turning radius, and high climbing ability compared to a wheel rail train [1]. The Changsha maglev express line launched tests on 26 December 2015 and officially began operation on 6 May 2016. For the electromagnetic maglev train system, suspension control is one of the key technologies [2]. The goal of suspension control is to ensure that the displacement between the electromagnet and the rail maintains a set value (such as 8 mm), providing a contactless magnetic support for the vehicle and achieving approximately zero height flight with no wheels.

It is worth noting that there is a lot of uncertainty in the number of passengers at each station. For example, the train is heavy and full at rush hour while it is in light-load conditions at non-peak times. When the load mass of a maglev train changes greatly, the suspension control system should be able to tolerate a wide range of change in the system parameters. In general, the load mass change can be regarded as a kind of internal disturbance [3,4]. Cui et al. [5] list several major factors of the carrying capacity of maglev trains, and analyze these influences on the lateral suspension force and horizontal guidance force to the suspension system. Lei et al. [6] point out that the load disturbance may make the system unstable and sometimes dangerous for passengers without an effective control strategy. Yau et al. [7] also introduce that when a maglev train passes over a flexible guideway with load variation, the vehicle interacts with the rail and self-excited oscillation occurs easily. These results show that when the load mass of the system changes greatly, the system performance will become worse and may even have self-excited vibration. Therefore, it is necessary to solve the problem of the stability of the suspension control of a maglev train with a large range of load disturbance.

Since frequent and large load changes are often encountered for a maglev system, any identification procedure should be able to, at least, detect load changes, or more positively, to find a suitable control method under load disturbance. If the parameters of the load mass can be acquired in real-time, the control system can update the system model, change the control parameters, and calculate a new control value in time to stabilize the system. However, for the maglev system, it is difficult to measure the load mass directly from the suspension control system under the existing conditions. The method of increasing the load mass or the number of force sensors for the whole train will not only significantly increase the cost of the control system, but also make the hardware system more complex. Currently, there is no simple and effective method for identifying the load disturbance of a maglev train directly [2,6].

Many research results reveal the reasons for the deterioration of the control system performance or the loss of stability when the load mass is significantly changed. Liu et al. [8] point out that a maglev system is a typical open-loop unstable system, and the relationship between levitation force, current, and the displacement of electromagnets shows strong nonlinear characteristics. In engineering, a nonlinear system is often linearized at its equilibrium point, and then the corresponding proportion-integral-derivative (PID) controller is designed [1,8]. When the load mass changes very little, the change of the equilibrium point of the suspension control system is also small. In this case, it can be regarded as an approximate linear system and the designed linear controller is usually effective. Once a system parameter of the maglev system, such as the load mass, changes significantly, the equilibrium point of the suspension system will shift considerably. In this situation, the stability ranges of the linear controller designed for a nonlinear maglev system are limited because the control parameters always remain unchanged. Thus, the control performance of the above linear controller will not be optimal or may even cause the system to lose stability when the load mass changes greatly [6].

So far, some researchers have proposed many methods to reject the load disturbance. One method is the linear gain scheduling method [9]. At first, the working area of the system can be divided into several small sub regions. For different sub regions, the subsystems are linearized and controllers with different equilibrium points are designed for each of them respectively, so that the control performance of the whole system is well-maintained in a large range. The problem of this method is that the more control laws, the more switching conditions. Additionally, a large number of control parameters need to be stored.

The second method is the nonlinear control method. In recent years, nonlinear system control technology, such as the phase plane method and the descriptive function method, has developed rapidly and has received extensive attention [10,11,12,13,14]. These methods take account of the nonlinear characteristics of systems, so the control performance using these methods is almost satisfied theoretically against system parameters that vary in a large range. However, in practical engineering practice, the stability of a nonlinear system cannot always be guaranteed all the time. For nonlinear maglev train systems, Sun et al. [10] establish a dynamic model without load disturbances and it is difficult to establish an exact mathematical model with load disturbances. Sadek et al. [11] present an adaptive fuzzy backstopping control method for a practical system with uncertain parameters, and the improved parameters are updated during each sampling period based on Lyapunov’s direct method. However, the uncertain dynamic of the system is approximated by a fuzzy system or a neural network, which can be further improved, and the complete proofs of stability also need to be provided. Kaloust et al. [12] design a nonlinear robust controller using nonlinear state transformation and Lyapunov's direct method in order to guarantee global stability for a nonlinear maglev system with uncertain dynamics, including negative damping due to eddy currents, but it is comprehensive and difficult to implement in hardware. Yang et al. [13] propose a robust controller for a nonlinear maglev system with mismatched uncertainties via a disturbance observer based control (DOBC) approach. However, the corresponding physical experiments have not been carried out, and whether the algorithm can be applied to a magnetic levitation system is still to be determined. Sun et al. [14] present a nonlinear dynamic model and a fuzzy sliding-mode controller for an electromagnetic levitation system of a low-speed maglev train; however, the complexity of the system and the difficulty of physical realization are increased. As for nonlinear differential equations, sometimes the solutions calculated by the theoretical method are far from the values of the actual system and it is not very accurate for analyzing the performance of nonlinear systems with these solutions [15]. Currently, there is no mature, universal method that can be used to analyze and design a variety of different nonlinear systems [1,10,14].

In view of the above problems, this paper focuses on the real-time adaptive control of a maglev train when the load mass is changed in a large range. First, the suspension control system model of a maglev train with a single electromagnet is set up and the open-loop stability of the system is analyzed. Then, a fast current-loop is used to simplify the control system and an adaptive control method is put forward, which ensures that the system is still in a stable state when the load mass of the train varies in a wide range. Finally, the effectiveness of the proposed method is verified by simulations and experiments.

## 2. Modeling of a Single Electromagnet System

**Figure 1:**
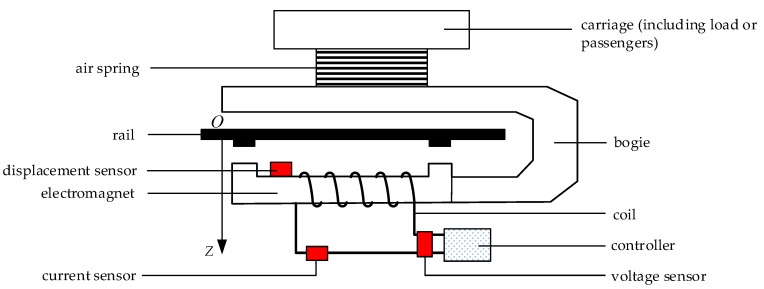
The composition of the suspension system of a single electromagnet of a maglev train.

When building up the mathematical model of a magnetic levitation system, if the details involved are too comprehensive, the order of the designed model will be very high [16]. The result is that the form of the control algorithm will be too complex, and it is not conducive to the specific implementation of the algorithm. There is no consideration of the influence of the air spring on the system [17], and it is assumed that the railway is rigid. Previous experiments show that under such assumptions, the designed controller can also make the system stable [10,15]. Figure 1 is a diagram of the suspension system of a single electromagnet of a maglev train.

As shown in Figure 1, the load or passengers are located inside the carriage, which is supported by the air spring. The air spring is fixed on the bogie, which is connected to the electromagnet. The displacement sensor is located at the upper part of the electromagnet and is used to measure the relative displacement between the electromagnet and the rail. The current and voltage of the electromagnet are measured by the current sensor and the voltage sensor, respectively. According to the displacement signal, the controller analyzes the movement speed of the electromagnet along the gravity direction, calculates an appropriate control value by the control algorithm, and outputs a corresponding current to the coil of the electromagnet. The electromagnetic attractive force between the electromagnet and the rail makes the electromagnet overcome the gravity of itself and the load mass and maintain a constant relative displacement between the electromagnet and the rail.

The attractive electromagnetic force of the electromagnet can be given as [15]

(1) $$F=k\frac{i^{2}}{z^{2}}$$

where $k=\frac{u_{0}N^{2}A}{4}$ is a constant about the air permeability $u_{0}$, the number of coil turns N, and the pole area A, i is the coil current of the electromagnet, z is the relative displacement between the electromagnet and the rail, and OZ is the direction of gravity.

For a maglev train system, the frequency of the electromagnet is almost greater than 20 Hz. In order to improve the comfort of the passengers (or the load), an air spring is selected with a low natural frequency of about 1 Hz to reduce the electromagnet force from the magnet to the carriage. Because the load of a maglev train system is above the air spring, the magnetic force of the electromagnet applied to the load changes very slowly by the isolation of the air spring. Therefore, when designing the suspension control system, the influence of the characteristics of the air spring on the dynamic characteristics of the whole maglev system can be neglected [16]. Within a short control period, the load mass or external disturbance can be considered to be approximately invariable.

The dynamic equation of the electromagnet is

(2) $$m\ddot{z}=(m+M)g-F＋f$$

where $\ddot{z}$ is the two order derivative of z, m is the mass of the electromagnet, M is the load mass above the air spring, *g* is the acceleration of gravity, F is the attractive electromagnetic force between the electromagnet and the rail, and f is the disturbance force above the air spring.

The voltage of the electromagnet is as follows

(3) $$u=R\cdot i+\frac{d\left(L\cdot i\right)}{dt}$$

where u is the voltage of the electromagnet, R is the resistance of the coils of the electromagnet, L is the equivalent inductor of the coil, and $L=\frac{2k}{z}$.

Then,

(4) $$u=Ri+\frac{2k}{z}\dot{i}-\frac{2ki}{z^{2}}\dot{z}.$$

where $\dot{z}$ is the derivative of z.

Denote the state of the system as x=(z,v,i). From Equations (2) and (4), the suspension system model of a single electromagnet of a maglev train can be derived as [8,12].

(5) $$\left\{ \begin{array}{l} \dot{z}=v\\ \dot{v}=\frac{(m+M)g}{m}-\frac{ki^{2}}{mz^{2}}+\frac{f}{m}\\ i^{'}=-\left(\frac{z}{2k}R-\frac{v}{z}\right)i+\frac{z}{2k}u \end{array}\right.$$

## 3. Fast Current Loop Design

### 3.1. Fast Inner Current-Loop Design

From Equation (5), we can find out that it is a typical three-order system. In order to simplify the analysis and design of the control system, it is necessary to reduce the order of the system. In the maglev system, the regulation speed of the current signal is much faster than that of the displacement signal. Therefore, the current control inner loop and the displacement control outer loop can be designed separately [8,18].

Because the current loop is composed of a resistance and an inductance, it can be regarded as a first-order link. In the small range of the electromagnet’s motion, the inductance is assumed to be a constant. Then, the system model is changed as [8]

(6) $$\left\{ \begin{array}{l} \dot{z}=v\\ \dot{v}=\frac{(m+M)g}{m}-\frac{ki^{2}}{mz^{2}}+\frac{f}{m}\\ i^{'}=-\frac{R}{L_{0}}i+\frac{u}{L_{0}} \end{array}\right..$$

The Laplace transform is obtained for the third equation of Equation (6) as follows

(7) $$\frac{i(s)}{u(s)}=\frac{1}{L_{0}s+R}.$$

**Figure 2:**
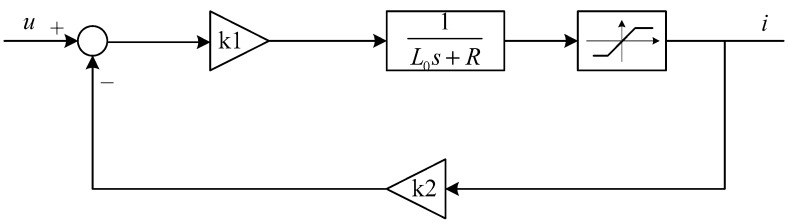
The control scheme of the fast current loop.

The current loop is designed as shown in Figure 2. In Figure 2, $k_{1}$ is the gain of the forward channel and $k_{2}$ is the gain of the reverse channel. 

From Figure 2, the closed-loop transfer function of the current loop can be obtained as

(8) $$\frac{i(s)}{u(s)}=\frac{k_{1}}{L_{0}s+R+k_{1}k_{2}}=\frac{\frac{k_{1}}{L_{0}}}{s+\frac{R+k_{1}k_{2}}{L_{0}}}.$$

As the bandwidth of the current loop is low, a proportional link with a gain of 1 can be designed at low frequency.

(9) $$\frac{i(s)}{u(s)}\approx 1$$

From Equations (8) and (9), we can get at low frequency

(10) $$\frac{k_{1}}{R+k_{1}k_{2}}=1.$$

Suppose $L_{0}=0.174,R=0.5$; then, the transfer function of the open current loop is

(11) $$\frac{i(s)}{u(s)}=\frac{1}{0.174s+0.5}.$$

Let $k_{1}=220,k_{2}=0.9977$. The transfer function of the closed current loop is then designed as

(12) $$\frac{i(s)}{u(s)}=\frac{220}{0.174s+220}.$$

**Figure 3:**
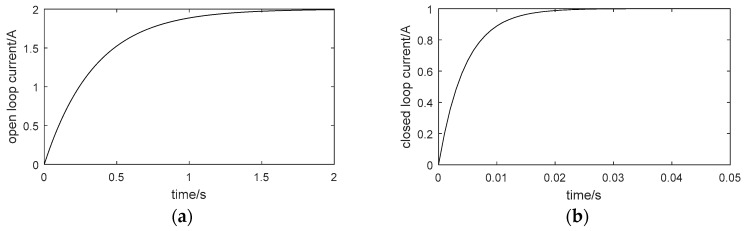
The step response curve of the inner current loop: (**a**) the open current loop and (**b**) the closed current loop.

Under ideal circumstances, the control voltage is not limited. If the gain is large enough, a smaller closed-loop time constant can be obtained. The ideal cases of simulation curves of the step response of the open current loop and the closed one are shown in Figure 3a,b respectively.

From Figure 3, the rise time of the open current loop is about 0.8 s while that of the closed current loop is 0.01 s. It can be seen that the regulation speed of the closed current loop is accelerated. At a low frequency, the corrected current loop can be equivalent to a proportional link with a gain of 1. It should be noted that it is just the result of an ideal simulation case and limited control voltage is not considered here.

### 3.2. Extraction of the Steady Current Signal

If the load mass changes, the steady-state current and the steady-state displacement of the electromagnet of the suspension system also change. It is assumed that the steady-state displacement of the electromagnet is fixed to be 8 mm; then, the steady-state current changes under different load conditions. We can only consider the variation of the steady-state current and estimate the present load mass.

The change of the load is very slow, while the current signal of the electromagnet measured by the current sensor changes quickly. Therefore, it is necessary to extract the direct current (DC) component of the current signal. The steady-state suspension current of the electromagnet can be adopted as the DC component.

Here, a traditional second-order resistance capacitance (RC) filter is selected to extract the DC component of the current signal. The transfer function of the low pass filter is

(13) $$H(s)=\frac{1}{1+RCs+2RC+R^{2}C^{2}s^{2}}$$

where *R* = 33 kΩ and *C* = 0.47 uF.

**Figure 4:**
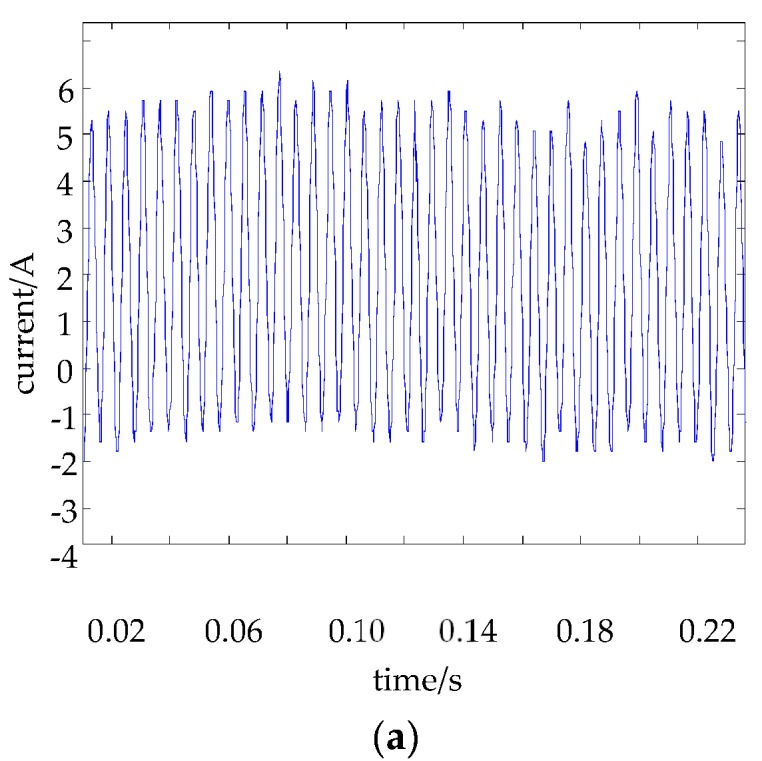

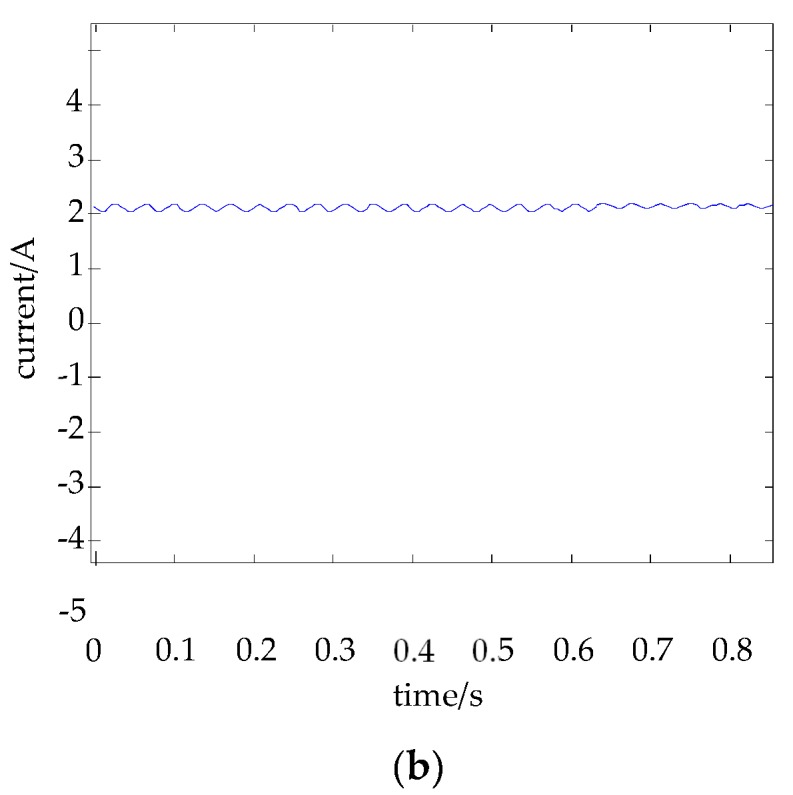
The curve of the electromagnet: (**a**) the real-time current from the current sensor and (**b**) the steady-state current obtained from the low pass filter.

Figure 4 is the curve of the real-time current of the electromagnet from the current sensor and the steady-state current obtained from the low pass filter (13).

Figure 4 further shows that the low pass filter can effectively filter the high-frequency components of the current signal and extract the low frequency effective value, which is approximate to the steady-state current value of the electromagnet.

## 4. Real-time Adaptive Control Law Design

**Figure 5:**
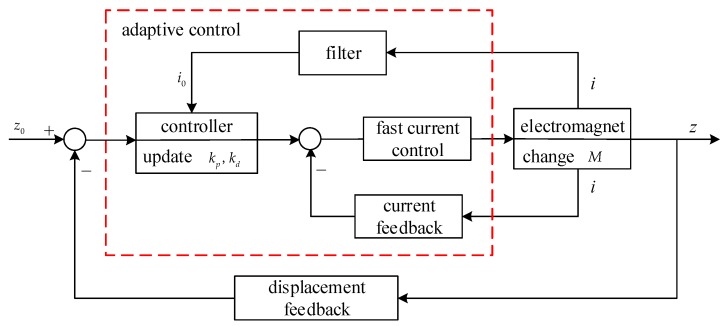
The diagram of real-time adaptive control of the maglev system with load disturbance.

After the above DC component value of the current is extracted, the adaptive control suitable for load mass change can be developed. The principle block diagram of the adaptive control is shown in Figure 5.

In Figure 5, when the load mass increases, the displacement between the electromagnet and the rail will increase immediately by gravity. Because there is not enough current to suspend the carriage with the increasing load any more, the controller has to output a greater control value as the current control input and the current of the electromagnet becomes larger by the fast current control. Then, the filter extracts the DC component of the current signal to the controller. The controller updates the control parameters adaptively and outputs a suitable current to the fast current control unit. Finally, the electromagnet provides a corresponding magnetic attractive force to suspend the carriage with the increasing load to the given displacement balance position.

### 4.1. Stability Analysis of the Reduced-Order System

In an experiment, the settling time of the displacement signal is generally about 0.15 s. Therefore, the speed of the current loop is much faster than that of the displacement loop [19,20]. By ignoring the delay effect of the current loop, the system is reduced to a two-order system and its system model is as follows [8]:

(14) $$\left\{ \begin{array}{l} \dot{z}=v\\ \dot{v}=\frac{(m+M)g}{m}-\frac{ku^{2}}{mz^{2}}+\frac{f}{m} \end{array}\right..$$

The equilibrium point of the reduced order system is 

(15) $$z_{0}=i_{0}\sqrt{\frac{k}{mg+Mg+f_{0}}},\;v_{0}=0.$$

Near the equilibrium point (15), the system (14) is linearized as:

(16) $$\left\{ \begin{array}{l} \dot{z}=v\\ \dot{v}=\frac{2(mg+Mg+f_{0})}{mz_{0}}z-\frac{2}{mz_{0}}\sqrt{(mg+Mg+f_{0})k}u \end{array}\right..$$

The system matrix of the linear system (16) is as follows:

(17) $$A=\left[\begin{array}{cc} 0 & 1\\ \frac{2(mg+Mg+f_{0})}{mz_{0}} & 0 \end{array}\right],B={\left[\begin{array}{cc} 0 & -\frac{2}{mz_{0}}\sqrt{(mg+Mg+f_{0})k} \end{array}\right]}^{T},C=\left[\begin{array}{cc} 1 & 0 \end{array}\right].$$

The characteristic equation of the two-order system is obtained as:

(18) $$\left|\lambda I-A\right|=\lambda^{2}-\frac{2(mg+Mg+f_{0})}{mz_{0}}.$$

From Equation (18), we can know that the two-order system is unstable. The controllable matrix of the two-order system is obtained from Equation (17):

(19) $$V_{c}=\left[\begin{array}{cc} 0 & -\frac{2}{mz_{0}}\sqrt{(mg+Mg+f_{0})k}\\ -\frac{2}{mz_{0}}\sqrt{(mg+Mg+f_{0})k} & 0 \end{array}\right].$$

It can be concluded that:

(20) $$rank(V_{c})=2.$$

It can be seen that the two-order system (14) is controllable.

### 4.2. Adaptive Control Law Design

For the magnetic levitation system, the most frequently used method is the state feedback control algorithm [1,9]. When this method is used, the control law of the system is expressed as:

(21) $$k_{p}\left(z-z_{0}\right)+k_{d}v.$$

The corrected system model is as follows:

(22) $$\left\{ \begin{array}{l} \dot{z}=v\\ \dot{v}=\frac{2(mg+Mg+f_{0})}{mz_{0}}z-\frac{2}{mz_{0}}\sqrt{(mg+Mg+f_{0})k}[u+k_{p}\left(z-z_{0}\right)+k_{d}v] \end{array}\right..$$

The state equation of the system (22) is

(23) $$A=\left[\begin{array}{cc} 0 & 1\\ \frac{2(mg+Mg+f_{0})}{mz_{0}}-Nk_{p} & -Nk_{d} \end{array}\right],\;B={\left(\begin{array}{cc} 0 & -N \end{array}\right)}^{T},\;C=(\begin{array}{cc} 1 & 0 \end{array})$$

where $N=\frac{2}{mz_{0}}\sqrt{(mg+Mg+f_{0})k}$.

The characteristic equation of the matrix A is:

(24) $$\left|\lambda I-A\right|=\lambda^{2}+Nk_{d}\lambda +Nk_{p}-\frac{2(mg+Mg+f_{0})}{mz_{0}}.$$

Thus, the range of control parameters of the two-order system can be obtained.

(25) $$k_{p}>\sqrt{\frac{mg+Mg+f_{0}}{k}},\;k_{d}>0$$

The transfer function of the corrected system is obtained by Equation (22):

(26) $$G(s)=C{\left(sI-A\right)}^{-1}B=\frac{-N}{s^{2}+k_{d}Ns+k_{p}N-\frac{2(mg+Mg+f_{0})}{mz_{0}}}.$$

The standard form of the transfer function of the two-order system is [8]

(27) $$G(s)=\frac{k_{a}\omega_{n}^{2}}{s^{2}+2\zeta \omega_{n}s+\omega_{n}^{2}}$$

where $k_{a}$ is the gain of the system. 

If the dynamic performance of the corrected system is required to meet that of the standard form, we can compare the denominator term with Equations (26) and (27):

(28) $$\left\{ \begin{array}{l} k_{d}N=2\zeta \omega_{n}\\ \omega_{n}^{2}=k_{p}N-\frac{2(mg+Mg+f_{0})}{mz_{0}} \end{array}\right..$$

Here, the overshoot of the design system is 3% and the settling time is 0.15 s. So

(29) $$\left\{ \begin{array}{l} e^{-\frac{-\pi \zeta }{\sqrt{1-\zeta^{2}}}}=0.03\\ \frac{4}{\zeta \omega_{n}}=0.15 \end{array}\right..$$

Thus, we can find out:

(30) $$\left\{ \begin{array}{l} \zeta =0.7448\\ \omega_{n}=35.8036 \end{array}\right..$$

Let $N=\frac{2}{mz_{0}}\sqrt{(mg+Mg+f_{0})k}$. Then, the expression of the control parameters is obtained from Equations (28) and (30) as follows [8].

(31) $$\left\{ \begin{array}{l} k_{p}=\frac{640.95mz_{0}}{\sqrt{(mg+Mg+f_{0})k}}+\sqrt{\frac{\left(mg+Mg+f_{0}\right)}{k}}\\ k_{d}=\frac{26.67mz_{0}}{\sqrt{(mg+Mg+f_{0})k}} \end{array}\right.$$

Here, Equation (31) satisfies the condition (25), so the system is stable.

However, in the usual case, the load mass M or the external force $f_{0}$ cannot be measured directly. The parameter identification method of some theories is complex, and the calculation speed cannot meet the real-time requirement of a maglev train system.

### 4.3. Control Parameters Selection

As mentioned earlier, if the displacement of the electromagnet is fixed, the change of model parameters of a magnetic suspension system is mainly on the load mass M or the external force $f_{0}$. They come mainly from the above part of the air spring [17]. Because of the isolation effect of the air spring, the force changes slowly when it is applied to the electromagnet. Thus, in each control period, it can be considered that the load disturbance is a constant [21,22,23].

By Equations (14) and (16), we can obtain in the balance state:

(32) $$i_{0}=z_{0}\sqrt{\frac{mg+Mg+f_{0}}{k}}.$$

The following expressions can be obtained by Equations (30) and (31):

(33) $$\left\{ \begin{array}{l} k_{p}=\frac{640.95mz_{0}^{2}}{ki_{0}}+\frac{i_{0}}{z_{0}}\\ k_{d}=\frac{26.67mz_{0}^{2}}{ki_{0}} \end{array}\right..$$

A different load mass *M* or external disturbance force *f* corresponds to a different equilibrium current $i_{0}$. When the algorithm needs to be realized, it is worth studying how the current of the electromagnet can be selected as the steady state current. In Section 3.2, a filter for the real-time current signal is proposed. In engineering, in the stage of initial suspension, with the decrease of the displacement of the electromagnet, the current is slowly increasing. When the displacement of the electromagnet is close to the rated value and its fluctuation is no more than 2 mm, the average value of the current can be regarded as the steady-state current corresponding to the load mass or the external disturbance force. In Equation (33), $i_{0}$ is in the denominator. When the current value is close to zero, the calculation result of Equation (33) will be very large. Under normal suspension conditions, the current of an electromagnet is generally greater than 5 A. In order to avoid this situation, when the current is less than 5 A, the value is assumed to be 5 A in the control algorithm.

The calculation expression of the control parameters is as follows:

(34) $$\left\{ \begin{array}{l} k_{p}=\frac{640.95mz_{0}^{2}}{ki(t)}+\frac{i(t)}{z_{0}}\\ k_{d}=\frac{26.67mz_{0}^{2}}{ki(t)} \end{array}\right..$$

When the system is stable, the convergent values of the control parameters are shown as Equation (32).

It can be seen that the adaptive control algorithm is simple in form and small in calculation. The control chip can update the control parameters in each control cycle. Therefore, the adaptive control law can be applied to a real-time magnetic levitation system.

## 5. Simulation Analysis

The system parameters of a single electromagnet of a maglev train are shown in Table 1.

**Figure 6:**
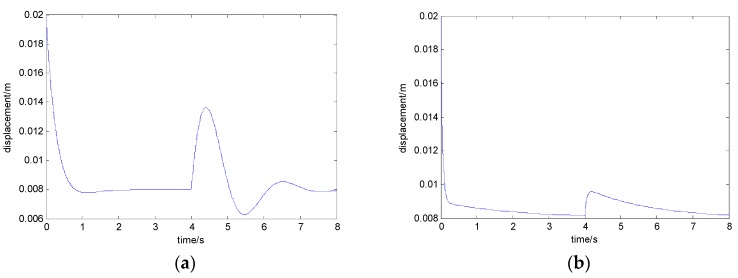
The curve of the electromagnet’s displacement when the load mass is increased by 200 kg: (**a**) with the state feedback control algorithm and (**b**) with the adaptive control algorithm.

**Figure 7:**
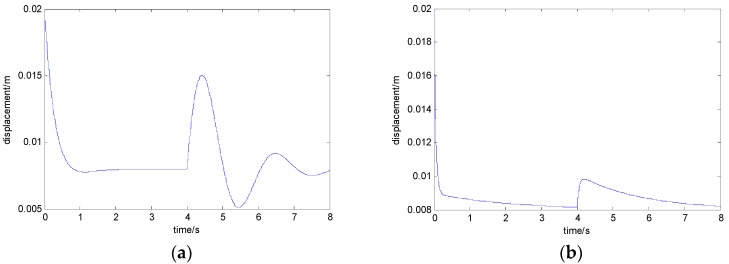
The curve of the electromagnet’s displacement when the load mass is increased by 450 kg: (**a**) with the state feedback control algorithm and (**b**) with the adaptive control algorithm.

**Figure 8:**
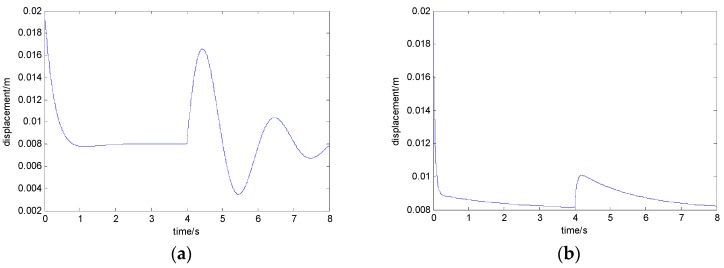
The curve of the electromagnet’s displacement when the load mass is increased by 700 kg: (**a**) with the state feedback control algorithm and (**b**) with the adaptive control algorithm.

When the load mass is increased by 200 kg, the curves of the electromagnet’s displacement with the state feedback control algorithm and the adaptive control algorithm are shown in Figure 6a,b, respectively. When the train load mass is increased by 450 kg, the curves of the electromagnet’s displacement with the state feedback control algorithm and the adaptive control algorithm are shown in Figure 7a,b, respectively. When the train load mass is increased by 700 kg, the curves of the electromagnet’s displacement with the state feedback control algorithm and the adaptive control algorithm are shown in Figure 8a,b, respectively.

From Figure 6, with the disturbance of a 200 kg load mass, if the adaptive control algorithm is not adopted, the maximum value of the electromagnet’s displacement is 13.9 mm, which exceeds the equilibrium position by 5.9 mm. When adopting the adaptive control algorithm, the maximum of the electromagnet’s displacement is 9.6 mm, which exceeds the equilibrium position by 1.6 mm.

From Figure 7, with the disturbance of a 450 kg load mass, if the adaptive control algorithm is not adopted, the maximum value of the electromagnet displacement is 15.1 mm, which exceeds the equilibrium position by 7.1 mm. When adopting the adaptive control algorithm, the maximum value of the electromagnet displacement is 9.9 mm, which exceeds the equilibrium position by 1.9 mm.

From Figure 8, with the disturbance of a 700 kg load mass, if the adaptive control algorithm is not adopted, the maximum value of the electromagnet displacement is 16.3 mm, which exceeds the equilibrium position by 8.3 mm. When adopting the adaptive control algorithm, the maximum value of the electromagnet displacement is 10.1 mm, which exceeds the equilibrium position by 2.1 mm.

It can be seen that when the load mass changes greatly, compared with the state feedback control method, the adaptive control method can make the fluctuation of the electromagnet displacement smaller, and the suspension performance remains unchanged.

**Figure 9:**
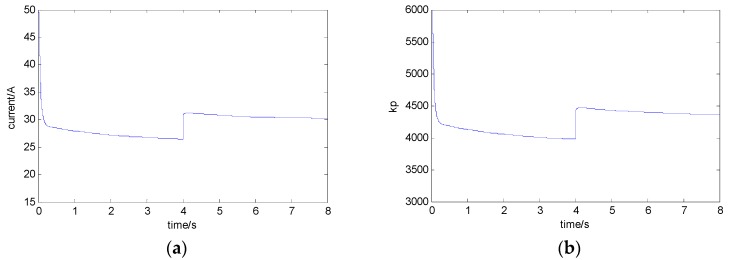

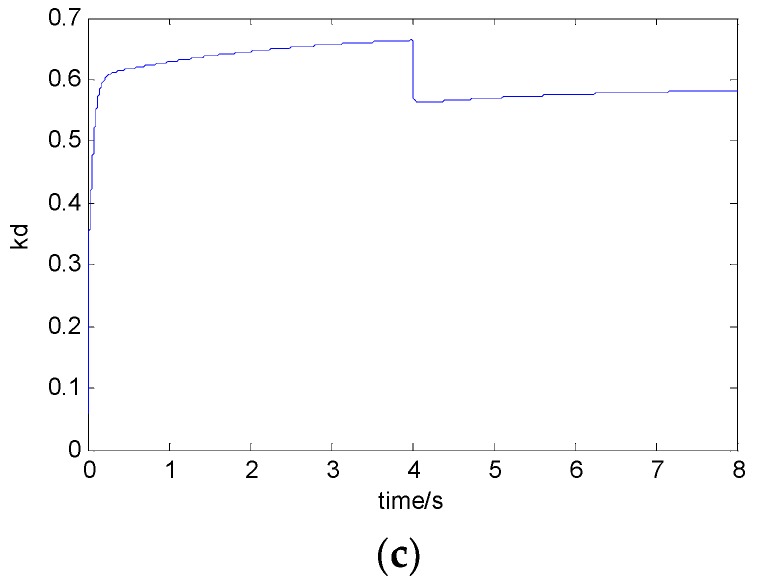
The curves of parameters: (**a**) the current of the electromagnet; (**b**) control parameter $k_{p}$; and (**c**) control parameter $k_{d}$.

In order to maintain the dynamic characteristics of the system, the corresponding control parameters should be adjusted according to the adaptive control method mentioned above in the case of the change of the load mass. With the different currents of different balance points, the curves of parameters $k_{p}$ and $k_{d}$ are as shown in Figure 9a–c.

As seen from Figure 9, after a steady current is obtained, the control parameters $k_{p}$ and $k_{d}$ can be adjusted online.

## 6. Experiment Results and Discussion

Light- and heavy-load conditions can be simulated by placing different weights on the carriage. The controller uses the system architecture of DSP and FPGA. Here, the DSP is the main processor, which is responsible for the calculation of the core algorithm and the output of PWM; FPGA is the coprocessor, which is responsible for the acquisition and filtering of the sensor signals and transmits the sensor signals to the DSP. The displacement sensor converts the analog voltage to a digital value and transfers it to FPGA, which makes it difficult to be interfered with in transmission. In order to eliminate the steady-state error of the system, the appropriate integral control is added to the algorithm, which can keep the relative displacement of the electromagnet and the rail near the equilibrium point.

**Figure 10:**
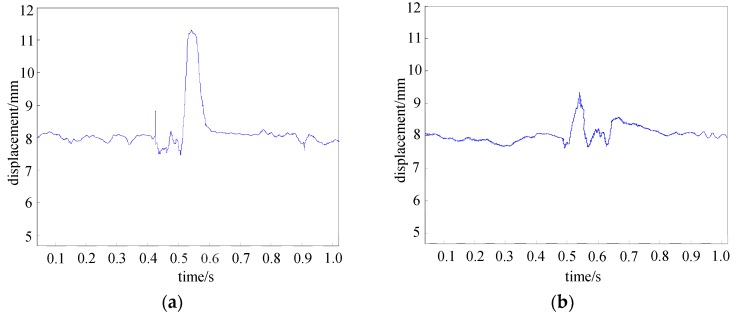
The curve of the electromagnet’s displacement when the load mass is increased by 250 kg: (**a**) with the state feedback control algorithm and (**b**) with the adaptive control algorithm.

The experimental results of the electromagnet’s displacement with the general state feedback method and the fast-adapting control method are given below under the condition of a wide range of load mass as shown in Figure 10.

In Figure 10a, the state feedback control algorithm is used for the suspension system. In 0.5 s, a load of 250 kg is increased above the electromagnet and the relative displacement of the electromagnet and rail is increased from 7.7 mm to 11.2 mm immediately. In 0.6 s, the relative displacement between the electromagnet and the rail is maintained at 8.1 mm. The maximum variation of the displacement signal is about 3.5 mm. In Figure 10b, the above adaptive control algorithm is adopted. In 0.5 s, a load of 250 kg is increased above the electromagnet and the relative displacement of the electromagnet and rail is increased from 7.8 mm to 9.5 mm immediately. In 0.6 s, the relative displacement between the electromagnet and the rail is maintained at 8.1 mm. The maximum variation of the displacement signal is about 1.7 mm. It can be seen that the fast-adapting control method designed in this paper can be used well in a magnetic levitation system.

## 7. Conclusions

In view of the wide range of the load mass of a maglev train, the suspension control performance deteriorates sharply and even loses its stability. In this paper, an adaptive control method for maglev systems considering a wide range of mass variation is presented. When the load mass changes, the relationship between the optimal control parameters and the steady-state current is deduced by obtaining the real-time steady-state suspension current. The control parameters are calculated online, and the adaptive control of the suspension system with load mass changes is realized. This method can ensure that the system is still in a stable suspension state when the load mass is changed in a large range. This is very beneficial for control parameter debugging for commercial systems.

## Figures and Tables

**Table 1:** The system parameters of a single electromagnet of a maglev train.

Term	Value	Unit
*k*	0.00545	N·m^2^/A^2^
*m*	725	kg
*R*	4.4	Ω
*z*_0_	0.008	m
*L*_0_	0.908	H
*g*	9.8	N/kg
